# Crosslinked Enzyme Aggregates (CLEAs) of Laccases from *Pleurotus citrinopileatus* Induced in Olive Oil Mill Wastewater (OOMW)

**DOI:** 10.3390/molecules25092221

**Published:** 2020-05-08

**Authors:** Anastasia Zerva, Christina Pentari, Evangelos Topakas

**Affiliations:** 1 InduBioCat Group, Biotechnology Laboratory, School of Chemical Engineering, National Technical University of Athens, 5 Iroon Polytechniou Str., Zografou Campus, 15780 Athens, Greece; anazer@chemeng.ntua.gr (A.Z.); pentari@chemeng.ntua.gr (C.P.); 2 Biochemical and Chemical Process Engineering, Division of Sustainable Process Engineering, Department of Civil, Environmental and Natural Resources Engineering, Luleå University of Technology, SE-97187 Luleå, Sweden

**Keywords:** crosslinked enzyme aggregates, *Pleurotus citrinopileatus*, laccase, olive oil mill wastewater.

## Abstract

The enzymatic factory of ligninolytic fungi has proven to be a powerful tool in applications regarding the degradation of various types of pollutants. The degradative potential of fungi is mainly due to the production of different types of oxidases, of which laccases is one of the most prominent enzymatic activities. In the present work, crude laccases from the supernatant of *Pleurotus citrinopileatus* cultures grown in olive oil mill wastewater (OOMW) were immobilized in crosslinked enzyme aggregates (CLEAs), aiming at the development of biocatalysts suitable for the enzymatic treatment of OOMW. The preparation of laccase CLEAs was optimized, resulting in a maximum of 72% residual activity. The resulting CLEAs were shown to be more stable in the presence of solvents and at elevated temperatures compared to the soluble laccase preparation. The removal of the phenolic component of OOMW catalyzed by laccase-CLEAs exceeded 35%, while they were found to retain their activity for at least three cycles of repetitive use. The described CLEAs can be applied for the pretreatment of OOMW, prior to its use for valorization processes, and thus, facilitate its complete biodegradation towards a consolidated process in the context of circular economy.

## 1. Introduction

Fungal species belonging to phylum Basidiomycota are often used for bioremediation/biodetoxification applications, due to their induced intricate enzymatic factory for the degradation of lignocellulosic biomass. Their potential for the biodegradation of organic pollutants lies in several major oxidative enzyme activities, mainly laccases (benzenediol:oxygen oxidoreductases, Lac, EC 1.10.3.2) and peroxidases, including manganese peroxidases (MnP, EC 1.11.1.13), lignin peroxidases (LiP, EC 1.11.1.14), and versatile peroxidases (VP, EC 1.11.1.16), among others [1]. These enzymes have been employed in the degradation of a wide range of pollutants, such as endocrine disrupting chemicals [2], dyes [3], pesticides [4], substituted phenols, pharmaceuticals [5], and others. Significant research efforts have been devoted to the degradation of olive oil mill wastewater (OOMW), the liquid byproduct of three-phase decanters of olive oil production plants. The disposal of OOMW poses a serious problem for olive-oil producing countries, since it is highly toxic to plants and soil-borne microorganisms, due to its high phenolic content, BOD, and COD values [6]. Many basidiomycetes are able to degrade OOMW almost completely [1,7,8,9], but the need for sterilization and the long incubation times required for fungal growth still impede the application of biological treatments as a feasible alternative to the currently used landfill deposition.

*Pleurotus* species are known to be potent degraders of phenol-rich contaminants [10,11], while their oxidative activity is mainly based on the production of multiple laccase enzymes [12], resulting in the production of high laccase titers during growth in phenol-containing media [1,13]. However, despite their high potential towards the oxidation of a wide variety of persistent pollutants, the enzymes derived from basidiomycetes usually are characterized by a low tolerance against extreme temperatures and pH, as well as the presence of solvents and inhibitors, as shown by numerous efforts to enhance their stability [14,15,16]. All these properties result in low stability of the enzymes in industrial process conditions.

Immobilization of biocatalysts is one of the most popular approaches to circumvent instability issues, offering the added advantage of reusability [17]. An immobilized catalyst with improved stability and recyclability properties can significantly lower the cost of the process. Moreover, immobilized enzymes can be used for the design of continuous processes, since the catalyst can be separated from the reaction mixture by a simple filtration or centrifugation step [18,19]. Among the various immobilization methods described in the literature, crosslinked enzyme aggregates (CLEAs) technology is one of the most prominent [20]. Many different enzymes have been immobilized in CLEA form, among them esterases [21], lipases [22], cellulases [23], and xylanases [24], which were used for various bioconversion applications, such as the production of cosmeceuticals [21], nutraceuticals [24], or even biofuels [22]. The advantages of CLEA technology include the simplicity of the method, the low overall cost of the procedure, and the fact that there is no need for solid support or dedicated specialized equipment. A possible disadvantage of the method is that it requires detailed optimization for each specific enzyme target, since the success of CLEA formation depends on the physicochemical characteristics of each enzyme. Throughout the years, CLEA technology has evolved to more refined and specialized techniques, such as combi-CLEAs [25], magnetic CLEAs (mCLEAs, [26]), porous CLEAs [27], and others [28], offering many choices for the tailored immobilization of any given enzymatic preparation.

Especially for laccases, many efforts have been made for their immobilization in CLEA form, and their consecutive use for biocatalytic applications. Some of the most recent efforts include the preparation of laccase CLEAs and their application to the decolorization of triphenyl methane and reactive dyes [29], the development of laccase-containing combi-CLEAs for the degradation of pharmaceuticals [30], and the immobilization of laccase in combi-CLEAs and their application in acetaminophen degradation [19]. For dye decolorizations, other CLEAs prepared from various laccases have also been employed, such as from *Pleurotus ostreatus* [29], *Trametes versicolor* [31,32], *Fomes fomentarius* [31], and *Cerrena* spp. [33].

In the present work, the crude enzymatic preparation from *Pleurotus citrinopileatus* grown in OOMW was immobilized in CLEAs. The formation of CLEAs was optimized based on three different parameters, and the resulting CLEAs were characterized as regards their laccase activity. As shown in our previous work, *P. citrinopileatus* is able to grow on OOMW as the sole carbon source and produce high titers of laccase activity, almost 1.5 U mL^−1^, while decolorizing the medium and decreasing the phenolic content up to 90%. In the same study, it was shown that the fungal biomass produced during OOMW degradation contained only traces of phenol compounds but significant quantities of β-glucans, which are bioactive compounds with numerous applications in cosmetics and dietary supplements [1]. In a follow-up study, bioreactor fermentation technology was employed, as well as different isolation protocols for the effective valorization of the biomass in the production of bioactive β-glucans [34]. In the present study, the OOMW-induced enzymatic system of *P. citrinopileatus* was immobilized, and the produced biocatalyst was utilized to a continuous process for the removal of the phenolic part of the waste material.

## 2. Results and Discussion

### 2.1. Immobilization of Crude Laccase Extract from OOMW-Grown P. citrinopileatus in CLEAs

**Figure 1:**
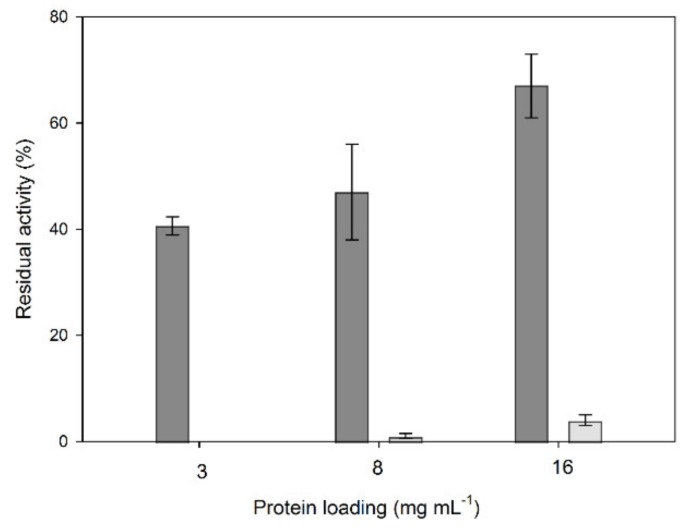
Effect of protein loading on the residual activity of crude laccase crosslinked enzyme aggregates (CLEAs). Dark grey bars: CLEAs, and light grey bars: residual free enzyme.

The first experiment towards the preparation of crude laccase CLEAs was the determination of the optimum protein loading in order to obtain satisfactory yields in terms of immobilization but also avoid leaching and, therefore, loss of the enzyme. Protein precipitation was performed with ammonium sulfate, and glutaraldehyde was used as a crosslinker. The activity was measured in the resulting CLEAs but also in the supernatant after CLEAs washing to measure the unbound laccase. The results are shown in Figure 1.

As shown in Figure 1, the highest residual activity in CLEAs was obtained in the highest protein loading tested. This result could be expected, given that the crosslinker concentration was kept constant, and therefore, in low protein concentrations, the crosslinker excess could lead to bigger aggregates, exhibiting more prominent diffusion limitations. Moreover, the unbound enzyme was higher with a higher protein loading, but still, the residual free enzyme in the highest protein loading was about 5.5% ± 0.6% of the CLEA-immobilized enzyme; therefore, the subsequent experiments were performed with the highest protein loading (16 mg mL^−1^).

**Figure 2:**
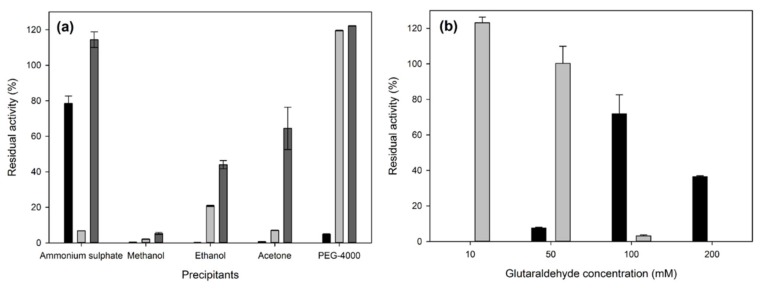
(**a**) Effect of precipitants on the formation of CLEAs. Glutaraldehyde was added as a crosslinker at a final concentration of 100 mM. Black bars: CLEAs, light grey bars: residual free enzymes, and dark grey bars: enzyme activity after precipitation and reconstitution (without the crosslinking step). (**b**) Effect of glutaraldehyde concentration on the formation of CLEAs. Saturated ammonium sulfate was used as the precipitant. Black bars: CLEAs, and light grey bars: residual free enzymes.

The first step in the preparation of the CLEAs is the precipitation of the desired enzyme. The selection of a suitable precipitant is of key importance in the success of immobilization, since the selected compound must induce precipitation effectively but also not be harmful to the structure of the enzyme. In the present study, five precipitants were tested, and the results are shown in Figure 2a.

As shown in Figure 2a, organic solvents, such as methanol, ethanol, and acetone, have a detrimental effect on the activity of the final immobilized enzyme. The maximum activity after precipitation and reconstitution of the protein preparation was observed in the case where ammonium sulfate or PEG-4000 was used as the precipitant. However, in the case of PEG-4000, the precipitated enzyme was not immobilized in CLEA form after the addition of glutaraldehyde. Therefore, ammonium sulfate was chosen as the precipitant for subsequent experiments, yielding CLEAs with residual activity 78.5% ± 4.2%. Ammonium sulfate is often found to be the optimal precipitant for immobilization applications [29], and, similarly to the results presented in this work, it has been shown to lead to increased enzyme activity [35].

The next step in CLEA formation is the crosslinking step, where covalent bonds are formed, connecting the enzyme molecules with the addition of a crosslinker. The most popular crosslinker is glutaraldehyde, since it seems to be efficient for most proteins, but also, it is a low-cost bulk chemical. The concentration of the crosslinker is also of great importance, since low concentrations can lead to inefficient crosslinking and enzyme leakage, while too high crosslinker concentrations can lead to mass transfer limitations due to the formation of a very rigid and compact structure. In the present work, different glutaraldehyde concentrations were tested, and the results are shown in Figure 2b. As expected with increasing glutaraldehyde concentrations, the residual free enzyme concentration is decreasing. However, in the case of residual activities in CLEAs, the maximum residual activity was observed in 100 mM glutaraldehyde (72% ± 10%), which was used for subsequent experiments. The observed activity recovery is comparable, but slightly higher, to other fungal laccase CLEAs in the literature [19,30,33,36,37].

The preparation of crude laccase CLEAs was also tested in two different scale reactions. Specifically, the immobilization of crude laccases in CLEAs was performed in a 10-μL and in a 100-μL initial sample volume, and the residual activity was measured. The activity recovery in the obtained CLEAs from the 100-μL sample reached 38.5% ± 2.6%. This is significantly lower than the activity recovery obtained with the 10-μL sample (72% ± 10%), probably due to different agitation conditions usually arising in the mixing of larger volumes but also due to the increased formation of larger aggregates during CLEA isolation by centrifugation. The loss of activity during the scale-up of the immobilization process could be circumvented in traditional or alternative reactor configurations, as in case of the perfusion basket reactor [38]. Such reactor configurations offer the added advantage of avoiding centrifugation of CLEAs, which can lead to the formation of larger aggregate clusters with severe mass diffusion limitations [28].

### 2.2. Characterization of Laccase CLEAs

**Figure 3:**
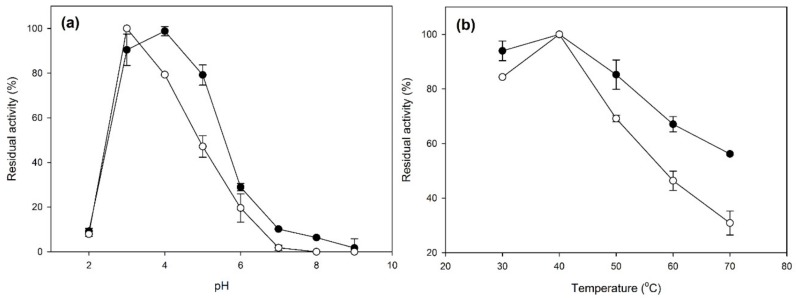
Effects of the pH (**a**) and temperature (**b**) on the activity of crude laccase CLEAs (white circles) and crude free enzymes (black circles).

For the characterization of the obtained CLEAs, the optimum activity conditions were determined. The results are shown in Figure 3.

As shown in Figure 3, the optimum pH for activity was slightly shifted in the case of immobilized laccases, from 4 to 3. This shift in the optimum pH is often observed in CLEA-bound laccase, and it is attributed to the conformation received by the enzyme at the microenvironment formed by the specific immobilization conditions [19,30,39,40]. The optimum temperature for laccase activity remained the same (40 °C) in the case of free and immobilized laccase, while in the case of laccase-CLEAs, the activity dropped more abruptly in temperatures higher or lower than the optimum. This might be due to the rigid conformation received by the enzyme molecule during immobilization, as well as the different microenvironment shaped by the crosslinker and the co-immobilized proteins [30]. Moreover, mass transfer limitations may affect the catalytic cycle [28] due to the short time of the enzymatic assay (10 min).

**Figure 4:**
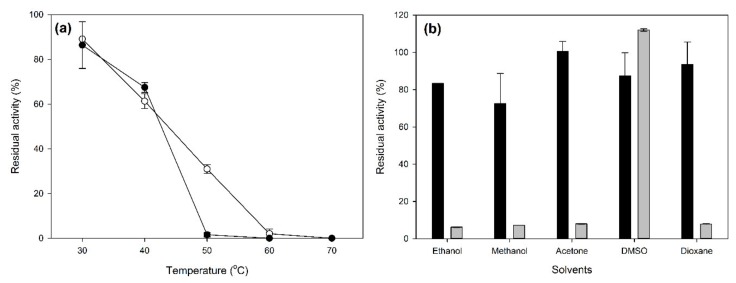
Effects of temperature (**a**) and organic solvents (**b**) on the stability of crude laccase-CLEAs. The residual laccase activity was measured after 24-h incubation in the indicated conditions. The calculation of residual activity was performed against suitable controls. White circles: CLEAs, black circles: crude free enzyme, black bars: CLEAs, and light grey bars: crude free enzymes.

The thermostability of the obtained CLEAs was also examined, and the results are shown in Figure 4.

As shown in Figure 4a, the laccase CLEAs showed a slightly improved thermostability compared to the free crude enzymes. More specifically, the difference is more prominent after incubation at 50 °C for 24 h, where the free crude laccase showed almost no activity, while the laccase CLEAs showed a residual activity of over 30%. The immobilization of enzymes in the form of CLEAs usually leads to a significant increase in thermostability [29,30,33,36]. The stability of free and immobilized laccase in the presence of solvents is shown in Figure 4b. Immobilization in CLEAs significantly enhanced the laccase stability in the presence of most tested solvents, since the free laccase was only stable in the presence of DMSO. Similar to thermostability, the increase in stability against organic solvents is common for enzymes immobilized in CLEAs [19,39,40] and is probably due to increased biocatalyst rigidity.

### 2.3. CLEA Application on OOMW Phenols Removal

**Figure 5:**
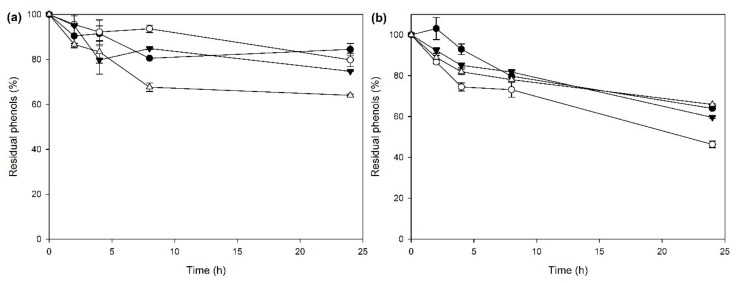
Effects of initial phenol concentrations at olive oil mill wastewater (OOMW) phenol removal by (**a**) laccase-CLEAs and (**b**) free crude laccase. White triangles: 0.324 g L^−1^ phenols, black inverted triangles: 0.646 g L^−1^ phenols, white circles: 1.8 g L^−1^ phenols, and black circles: 3.5 g L^−1^ phenols.

In the context of developing a consolidated process for the removal of OOMW phenols, laccase-CLEAs were applied on the treatment of OOMW in 24-h reactions. Aside from the obvious advantage of CLEA reuse, enzymatic treatment of OOMW also offers the possibility to use nonsterilized materials, as opposed to microbial treatment, which requires the sterilization of the medium. The time profile of phenol removals for the different OOMW concentrations tested is shown in Figure 5a. The highest concentration used (3.5 g L^−1^) corresponds to 90% (*v*/*v*) OOMW, with the addition of buffer pH 4.

As shown in Figure 5a, the removal of phenols for concentrations over 0.646 g L^−1^ reached up to 25% after 24 h. However, for 0.324-g L^−1^ phenols, the CLEA-mediated removal reached almost 36% phenol removal. The respective results for the nonimmobilized crude laccase are shown in Figure 5b. Although the same units of laccase activity were added to both experiments, in the case of free crude laccase, a higher degree of phenol removal was observed, over 50%. A possible explanation for this observation is that the oxidation of OOMW phenols might lead to polymerization and precipitation of the oxidation products, with deleterious effects for the enzyme activity of the insoluble biocatalyst. In order to check this hypothesis, laccase-CLEAs were applied to the removal of OOMW phenols in an 8-h reaction, and after that, the CLEAs were removed with centrifugation, washed three times, and applied again to the same material. For the first cycle, the removal of phenols reached 16.8% ± 0.5%, while, after the second cycle, an additional 0.95% ± 0.02% was removed. These results indicate that laccase-CLEAs possibly have a narrower substrate range in regard to OOMW phenols compared with the free laccases, since after washing, they are still unable to remove further the phenolic content of OOMW. Other oxidative enzyme activities were also measured, assuming that, through the immobilization process, laccase activity was enriched, and therefore, other relevant enzymes for the efficient removal of a broader range of phenolics may have been removed. Indeed, the crude enzyme sample contained 3.1 ± 0.2 U mL^−1^ of manganese-independent peroxidase activity and 1.3 ± 0.26 U mL^−1^ of manganese-dependent peroxidase activity, while in the CLEA preparations, only 0.22 ± 0.01 U mL^−1^ of manganese-dependent peroxidase activity was detected (corresponding to 16.9% activity recovery), and manganese-independent peroxidase activities were completely abolished. In view of these results, we could assume a rather significant role for peroxidases in the phenol removal of OOMW, at least under the experimental conditions described here. Although hydrogen peroxide was not added to the reaction mixture, the existence and complementary activity of H_2_O_2_-producing oxidases can be assumed, such as aryl-alcohol oxidases and glyoxal oxidases. In order to support this hypothesis, veratryl alcohol-oxidizing activity was also measured in the crude enzyme mixture, and in the CLEAs, and the results were 0.043 ± 0.0 U mL^−1^ and 0.019 ± 0.0005 U mL^−1^, respectively, corresponding to 45% ± 5.4% activity recovery in the CLEA preparation.

**Figure 6:**
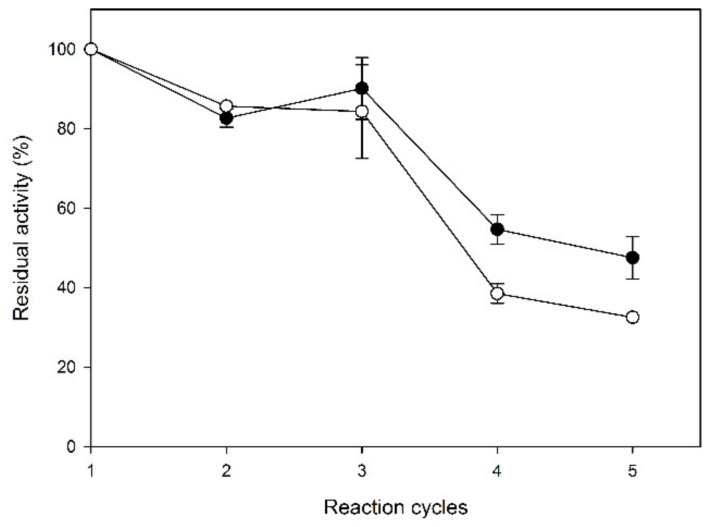
Operational stability of laccase-CLEAs in batches of 8-h reactions of OOMW removal. Black circles: CLEAs were washed between cycles, and white circles: CLEAs were used without washing.

Laccase-CLEAs were used in repetitive cycles of OOMW phenol removals, with or without washing, where fresh OOMW medium was added in each cycle in order to elucidate the possible inhibition of the catalysts by residual absorbed oxidized products. The results are shown in Figure 6.

In the first three cycles of OOMW treatment, washing did not seem to affect the activity of laccase-CLEAs, while they were shown to retain almost fully their activity (over 84% residual activity after the first three cycles of the OOMW treatment). After that, the residual activity dropped abruptly for both washed and not-washed CLEAs, and the difference between them became more prominent. After five cycles of repetitive OOMW treatment, the residual activity of washed CLEAs reached 47.6% ± 5.3%, while for nonwashed CLEAs, the residual activity was 32.5% ± 0.3%. The laccase activity was measured with ABTS (2,2′-Azino-bis(3-ethylbenzothiazoline-6-sulfonic acid)) as the substrate in the supernatants, after each reaction cycle, to determine the possible enzyme leaching from the CLEAs. Only the supernatants from the first reaction cycle were found to have traces of laccase activity, corresponding to 1.41% ± 0.05% of the initial, for the nonwashed CLEAs, and to 1.42% ± 0.15% of the initial for the washed CLEAs. After the first cycle, laccase activity was not detected in the supernatants.

In the literature, only a few studies report the enzymatic treatment of OOMW with oxidative enzymes. Justino et al. [41] showed varying degrees of phenol removal from OOMW (4–70%) with a commercial laccase from *T. versicolor* after treatment for seven days with a starting phenol concentration of 60.8 mg L^−1^, much lower than in the present study. Nugroho Prasetyo et al. [42] tested various laccases from different sources, supplemented with 1-hydroxybenzotriazole as the mediator, for the phenol removal of OOMW, with a starting concentration of 689 mg L^−1^. The application of laccase from *Trametes villosa* resulted in 64% phenol removal, while *Myceliophthora thermophila* laccase removed up to 32% in 24-h experiments. The authors explained this difference based on the different redox potentials of these laccases, resulting in a higher substrate range for laccases from *T. villosa* than that of *M. thermophila*.

## 3. Materials and Methods

### 3.1. Olive Mill Wastewater

OMWW was obtained from an olive oil mill with a three-phase decanter in Kalamata (Peloponnese, S.W. Greece) and maintained at −20 °C. The composition and pretreatment procedure were as reported in [1].

### 3.2. Microorganisms and Culture Procedures

*P. citrinopileatus* LGAM 28,684 was obtained from the fungal culture collection of the Laboratory of General and Agricultural Microbiology (Agricultural University of Athens, Greece). The strain was maintained in potato dextrose agar plates (PDA; Applichem, Darmstadt, Germany) at 4 °C. Solid precultures and liquid cultures were performed as described in [1]. After 12 days of growth in OMWW medium, the supernatant of the culture was collected by centrifugation and was concentrated 10 times by ultrafiltration before further analyses.

### 3.3. Enzyme Activities

Laccase activity was measured as described in [1], with 2-mM ABTS as the substrate in 35 °C. Peroxidase activities were measured according to the method of Ngo and Lenhoff [43] at 35 °C. Veratryl alcohol-oxidase activity was measured according to the authors of [10]. One unit of enzyme activity corresponds to the amount of enzyme releasing 1 μmol of product per minute in the above conditions. Protein concentration was determined using the Bradford method [44].

### 3.4. Immobilization of Crude Laccase Extract

The crude enzyme preparation was immobilized to CLEAs, as previously described [45]. Briefly, 10 μL of enzyme solution with 16% (*w*/*v*) protein content were added to 90 μL of the selected precipitant, followed by 15 min incubation at room temperature and 1200 rpm. Then, 4 μL of glutaraldehyde solution 2 M (100-mM final concentration) was added to the mixture, and the crosslinking was left to proceed for 3 h at room temperature and 1200 rpm. The mixture was quenched in 900-μL 100-mM potassium phosphate buffer, pH 6, and centrifuged. The supernatant was assayed for activity. CLEAs were washed twice with potassium phosphate buffer to remove unreacted enzyme and glutaraldehyde. Supernatants were also assayed for activity where no enzyme leakage was observed. Finally, the CLEAs were dispersed in 1-mL potassium phosphate buffer, and an aliquot was withdrawn for activity measurement. The scale-up of crude laccase-CLEAs was performed with 100-μL enzyme solution, 900-μL precipitant, and 40-μL glutaraldehyde solution.

### 3.5. Characterization of Laccase-CLEAs

The stability of the CLEA preparations in the presence of organic solvents was tested as follows: Crude enzyme preparation or CLEAs were added to a mixture of 50% (*v*/*v*) solvent in sodium phosphate buffer 100 mM, pH 7, at a final volume of 1 mL. The mixtures were incubated in 4 °C, under agitation for 24 h in an Eppendorf’s thermomixer (Eppendorf, Hamburg, Germany), and then, the activity was measured. The residual activity is expressed as a percentage of the remaining activity compared with the incubation of the appropriate amount of CLEAs or free enzymes in dH_2_O for 24 h at 4 °C. The stability of CLEAs at different temperatures was tested by adding the free or immobilized enzyme preparations in sodium phosphate buffer 100 mM, pH 7, and incubating for 24 h at different concentrations. The residual activity was expressed as a percentage of the remaining activity compared to the initial activity (t = 0).

### 3.6. Application of Laccase-CLEAs on OOMW Phenol Removal

For the removal of phenols from OOMW, 2 mg of laccase-CLEAs (prepared from the 50-μL crude enzyme sample) or 37 μL of the crude enzyme sample (corresponding to the same laccase units to the immobilized enzyme) were added to 1 mL of OOMW in different concentrations in phosphate-citrate buffer 100 mM, pH 4. The reactions were incubated in a shaking incubator at 35 °C, 200 rpm, and samples were withdrawn at selected time points. Samples were boiled, centrifuged, and the phenol content was measured with the Folin-Ciocalteu reagent [1]. Boiled CLEAs were used as controls in order to correct the results for the possible absorption of phenols, but phenol absorption in the boiled CLEAs was not observed. In order to determine the operational stability of the CLEAs, the OOMW phenol removals were performed in 8-h reactions in an OOMW medium with 1.8 g L^−1^ phenols in 100-mM citrate-phosphate buffer, pH 4. After each cycle, the CLEAs were removed by centrifugation, and they were used for a new reaction cycle with a freshly prepared medium with or without washing. The supernatant, after boiling for 10 min, was used for the quantification of the phenols. Aliquots from the supernatant were also used for the determination of the laccase activity with the ABTS assay, as described in Section 3.2.

## 4. Conclusions

In the present work, crude laccases from the culture supernatant of OOMW-grown *P. citrinopileatus* were concentrated and immobilized in CLEAs. The immobilization procedure was optimized, and the resulting CLEAs were characterized and eventually applied to the treatment of OOMW for phenol removal. The obtained laccase-CLEAs were shown to remove the phenolic content of OOMW up to 35%, while they retain over half of their activity for at least three cycles of reuse. The proposed system could potentially be applied in the enzymatic treatment of OOMW, removing the need for sterilization, which is an added advantage in comparison with microbial treatments. The immobilization of crude *P. citrinopileatus* laccases in the form of CLEAs, and their application in phenol removal from OOMW, could offer a viable solution for the valorization of the spent medium after fungal growth towards the production of valuable biocatalysts with bioremediation potential. Future implementation of the results of the present work could potentially include the production of combi-CLEAs, enriched with other oxidative enzymatic activities such as peroxidases, but also, the adaptation of the procedure to bioreactor scale a under continuous mode, with the aim to increase further the efficiency of the CLEA-mediated bioremediation of OOMW.
